# Failure load of the femoral insertion site of the anterior cruciate ligament in a porcine model: comparison of different portions and knee flexion angles

**DOI:** 10.1186/s13018-021-02676-z

**Published:** 2021-08-24

**Authors:** Shohei Yamauchi, Kyohei Ishibashi, Eiji Sasaki, Shizuka Sasaki, Yuka Kimura, Yasuyuki Ishibashi

**Affiliations:** 1 Department of Orthopaedic Surgery, Hirosaki University Graduate School of Medicine, 5 Zaifu-cho, Hirosaki, Aomori, 036-8562 Japan; 2 Hirosaki University School of Medicine, Hirosaki, Japan

**Keywords:** Anterior cruciate ligament, Biomechanics, Femoral insertion, Failure load, Anteromedial bundle, Posterolateral bundle, Direct insertion, Indirect insertion

## Abstract

**Background:**

This study compared the failure load of the femoral insertion site of the anterior cruciate ligament between different portions and knee flexion angles.

**Methods:**

In total, 87 fresh-frozen, porcine knees were used in this study. Three knees were used for histological evaluation; the remaining 84 knees were randomly divided into 4 groups: anterior anteromedial bundle, posterior anteromedial bundle, anterior posterolateral bundle, and posterior posterolateral bundle groups (*n*=21 per group). The anterior cruciate ligament femoral insertion site was divided into these four areas and excised, leaving a 3-mm square attachment in the center of each bundle. Tibia-anterior cruciate ligament-femur complexes were placed in a material testing machine at 30°, 120°, and 150° of knee flexion (*n*=7), and the failure load for each portion was measured under anterior tibial loading (0.33 mm/s).

**Results:**

Histological study showed that the anterior cruciate ligament femoral insertion site consisted of direct and indirect insertions. Comparison of the failure load between the knee flexion angles revealed that all the failure loads decreased with knee flexion; significant decreases were observed in the failure load between 30 and 150° knee flexion in the posterior anteromedial bundle and posterior posterolateral bundle groups. Comparison of the failure load according to different portions revealed a significant difference between the anteromedial and posterolateral bundle groups at 150° of knee flexion, but no significant difference among the groups at 30° of flexion.

**Conclusions:**

Although the failure load of the posterior portion decreased significantly in the knee flexion position, it (mainly consisting of indirect insertion) plays a significant role against anterior tibial load in the knee extension position; this appears to be related to the characteristics of the insertion site. Reflecting the complex structure and function of the ACL, this study showed that the failure load of the femoral insertion site varies with differences in positions and knee flexion angles.

## Background

The anterior cruciate ligament (ACL) is the most commonly injured ligament within the knee joint during sports activities. Although ACL reconstruction is the standard treatment for ACL injury with satisfactory results in most cases, postoperative stability is not necessarily perceived as subjective improvement [1]. The most frequent technical error is an anterior placement of the femoral tunnel [2]; another reason for the unsatisfactory results is that the geometric complexity of the ACL cannot be completely reproduced during surgical reconstruction [3]. Therefore, numerous biomechanical and anatomical studies have been conducted on ACL reconstruction to improve its results.

Based on macroscopic observations, the femoral ACL insertion point is relatively large and oval in shape at the medial surface of the lateral femoral condyle; it is located posterior to the lateral intercondylar ridge, which is also known as the resident’s ridge [4–8]. The ACL fibers are aligned parallel to the intercondylar roof in the fully extended position; the posterior portion of the femoral attachment consists of fanlike extension fibers that appear to be thin and coarse compared to the mid-substance fibers [8]. Histologically, the ACL femoral insertion site consists of direct and indirect insertions, and the posterior portion adjacent to the posterior articular cartilage margin shows indirect insertion [6–9]. Moulton et al. [9] revealed the detailed morphology of the human ACL femoral insertion site using scanning electron microscopy. The transition zone from the direct fibers to the bone had more interdigitations than that of the indirect insertion site; this may indicate the importance of direct insertion.

Several studies have investigated the topographical function of the ACL femoral insertion site using a 6-degree of freedom (DOF) robotic manipulator with a 6-axis force/torque sensor. Kawaguchi et al. [10] reported that the most important area of the ACL femoral insertion site was the central anterior part, near the roof of the resident’s ridge. Using a 6-DOF robotic manipulator, Nawabi et al. [11] also reported that high fibers of the femoral ACL insertion site (direct insertion) near the resident’s ridge carried a greater force than the low fibers (indirect insertion) in response to simulated Lachman, anterior drawer, and pivot shift tests. Pather et al. [12] showed that the contribution of indirect insertion was trivial against tibial translation and rotation. This was demonstrated in a physiological environment with the help of an arthroscope, using a robotic manipulator. These studies showed the biomechanical superiority of direct insertion over indirect insertion.

Recently, Sabzevari et al. [13] demonstrated the importance of the posterior fan-like extension of the femoral ACL insertion site in increasing the ligament failure load. However, the significance of tunnel placement at this location is clinically unclear. The purpose of this study was to compare whether the failure load of the femoral ACL insertion site differs in different portions and knee flexion angles in porcine knees. We hypothesized that the posterior portion (indirect insertion) of the ACL was as strong as the anterior portion (direct insertion) and that these strengths changed depending on the knee flexion angle.

## Methods

Eighty-seven fresh-frozen, mature porcine knees were used in this study. Specimens were stored at −20 °C until testing and thawed for 24 h prior to testing [14, 15]. This study was approved by the Institutional Review Board of the authors’ affiliated institutions.

### Histological evaluation

Three fresh-frozen porcine knees were used for histological evaluation of the ACL femoral insertion site. All soft-tissue structures around the knee were removed to expose the joint; the ACL was cut in the middle, and the knee joint was disarticulated. The femur was carefully cut in the sagittal plane by an oscillating saw through the uppermost point of the outlet of the intercondylar notch, avoiding damage to the ACL femoral insertion site [7]. These femur-ACL complex specimens were decalcified in K-CX solution (Falma, Tokyo, Japan) for 10 days depending on bone quality. The specimens were cut along the ACL fiber arrangement and embedded in paraffin after dehydration. Histologic sections (5-μm thick) were made parallel to the roof of the intercondylar notch and perpendicular to the bone surface [7, 8]. The sections were stained with hematoxylin and eosin to observe the morphology of the ACL insertion site. For histological analysis, a light microscope (All-in-one Fluorescence Microscope BZ-X700, Keyence, USA) was used at magnifications of 40×, 100×, and 400×.

### Tensile testing

**Fig. 1:**
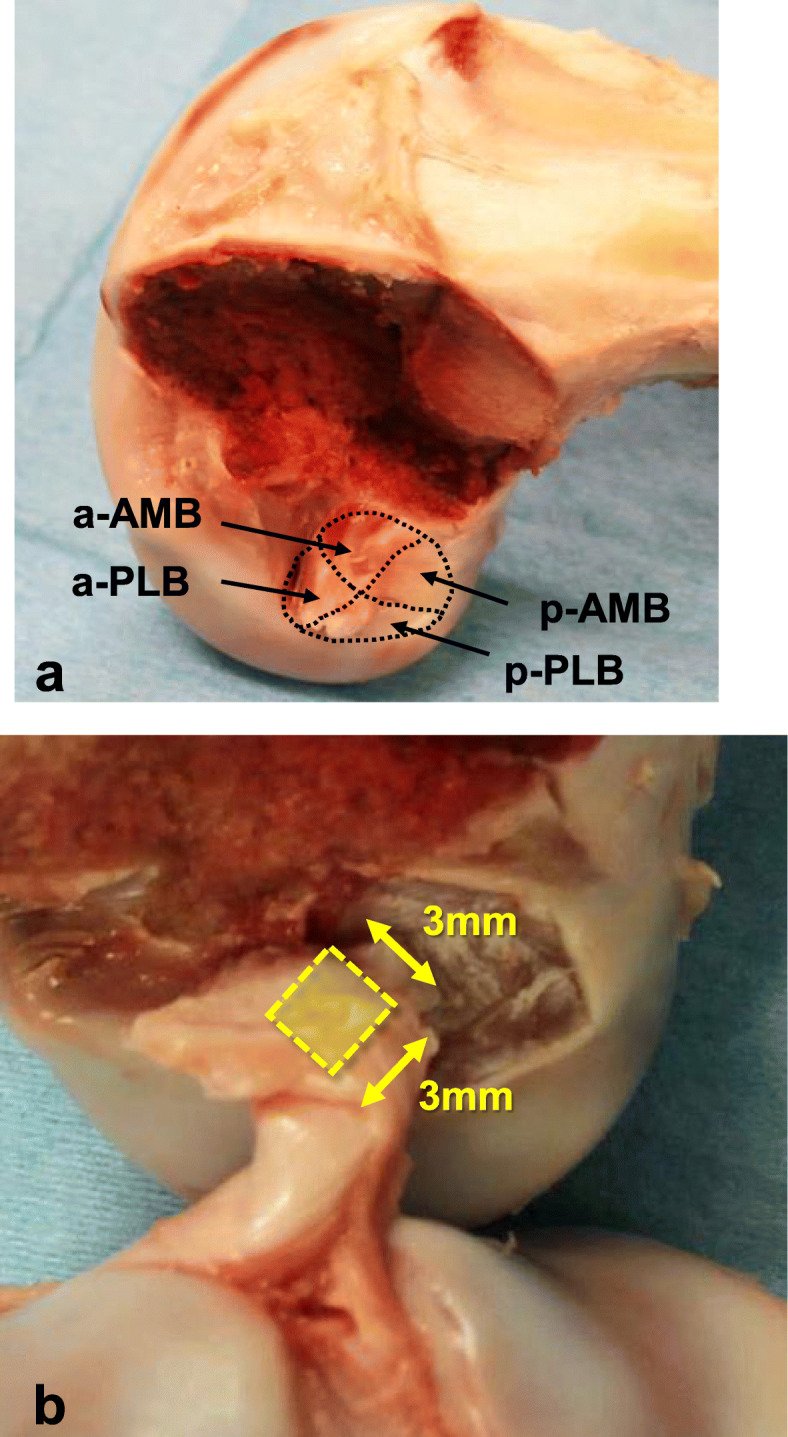
The porcine femoral ACL insertion site and classification of the insertion area. **a** The femoral ACL attachment is divided into the following 4 areas: anterior half of anteromedial bundle (AMB) (a-AMB), posterior half of AMB (p-AMB), anterior half of posterolateral bundle (PLB) (a-PLB), and posterior half of PLB (p-PLB). **b** The 3-mm square attachment in the center of each bundle was left for the pull-out tests. This photo shows the a-AMB group

Eighty-four fresh-frozen porcine knees were used for mechanical testing. The femur and tibia were cut approximately 100 mm from the joint line. All soft-tissue structures including the collateral ligaments and joint capsule around the knee were removed to expose the joint, and the soft-tissue structures within the joint, except the ACL, were also removed. The tibia and femur were potted in epoxy resins and fixed to a metal pot using several screws. The medial femoral condyle was resected using an oscillating saw to avoid damage to the ACL insertion site, and the femoral attachment of the ACL was observed. The femoral ACL attachment was divided into the following four areas: anterior half of the anteromedial bundle (AMB) (a-AMB), posterior half of the AMB (p-AMB), anterior half of the posterolateral bundle (PLB) (a-PLB), and posterior half of the PLB (p-PLB) (Fig. 1a). The 84 knees were randomly divided into 4 groups of 21 knees each. The ACL femoral attachment was divided into the abovementioned four areas and excised using a surgical number 11 blade, leaving a 3-mm square attachment in the center of each bundle (Fig. 1b). These femur-ACL-tibia complexes (FATC) were used for the pull-out tests.

**Fig. 2:**
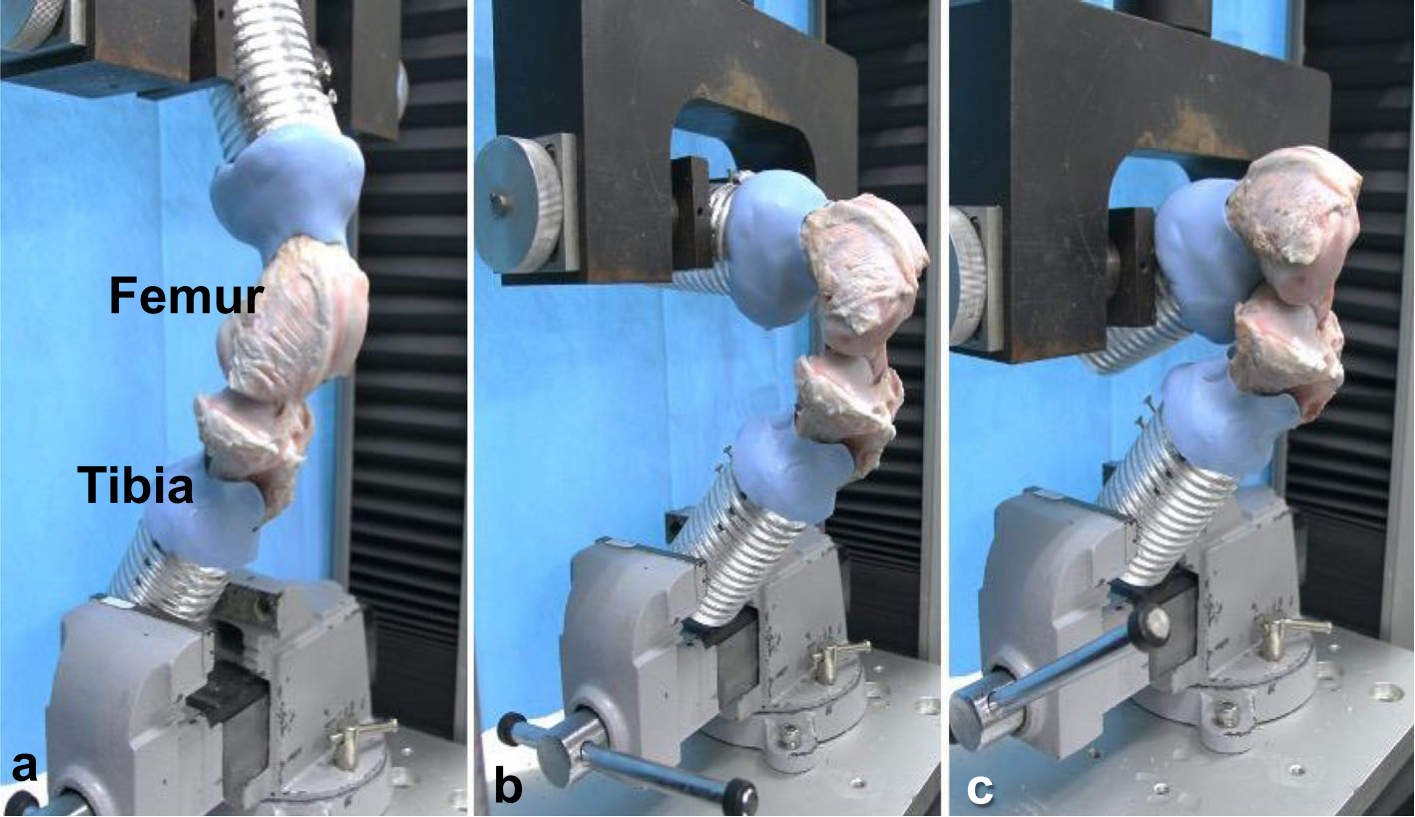
Testing apparatus and specimen (femur-ACL-tibia complex: FATC). FATCs were mounted on the material testing machine in the three knee flexion angles (**a** 30°, **b** 120°, and **c** 150°). The tibia was fixed to adjust the tibia angle so that the tensile load was applied along the axis of the ACL

The FATC samples were placed in a material testing machine (Instron 4465, Instron, Canon, MA) (Fig. 2). The tibia was fixed to the base of the testing machine at neutral rotation to adjust the tibia angle, so that the tensile load was applied along the axis of the ACL. The femur was fixed to the cross head of the machine to the knee joint angles of 30° (full extension), 120°, or 150° (7 knees in each). The FATCs of each group were pulled out vertically along with ACL fibers to failure at a rate of 0.33 mm/s [16]. The failure mode of each specimen was recorded; the failure load was defined by the maximum load, and the stiffness was defined by the load-displacement data [8]. In this study, no cyclic loading was applied to the knee.

### Statistical analysis

The failure load and stiffness of the four groups (a-AMB, p-AMB, a-PLB, and p-PLB), at knee flexion angles of 30°, 120°, and 150° have been presented as means and standard deviations. The results showed that the standard deviation of the failure load for the overall testing was 68.5 N. When 7 specimens were used in each group, post hoc power analysis revealed that the statistical power with 5% type I error was 0.884, and the effect size was calculated as 0.871.

The mean failure load and stiffness of the four groups at knee flexion angles of 30°, 120°, and 150° were compared by one-way analysis of variance, and the Tukey test was used for post hoc analysis. All data inputs and statistical calculations were performed using IBM SPSS Statistical Software (version 27.0, IBM Corp., Armonk, NY, USA). A *p*-value < 0.05 was considered statistically significant.

## Results

### Histological evaluation

**Fig. 3:**
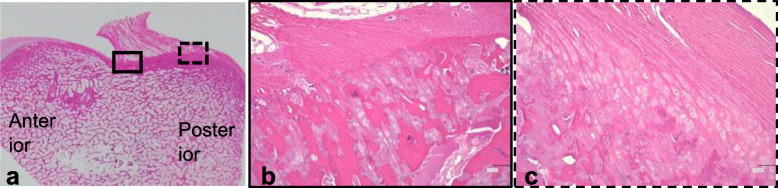
Histology of the porcine femoral ACL insertion site on hematoxylin and eosin staining. **a** On the medial surface of the lateral femoral condyle, the femoral anterior cruciate ligament (ACL) insertion site is located between the lateral intercondylar ridge (arrow head) and the posterior condyle cartilage margin (arrow). **b** The anterior attachment of the femoral ACL insertion site shows direct insertion. **c** The posterior attachment of the femoral ACL insertion site shows indirect insertion

On the medial surface of the lateral femoral condyle, the femoral ACL insertion site was located between the lateral intercondylar ridge and the posterior condyle cartilage margin (Fig. 3a). The anterior attachment of the femoral ACL insertion site showed direct insertion, which consisted of the ligament, noncalcified fibrocartilage, calcified fibrocartilage, and bone with tidemarks between noncalcified and calcified fibrocartilages (Fig. 3b). The posterior attachment of the femoral ACL insertion site showed indirect insertion, which consisted of a 2-layered structure where the ligaments were directly anchored to the bone by Sharpey-like fibers (Fig. 3c).

### Tensile testing

**Fig. 4:**
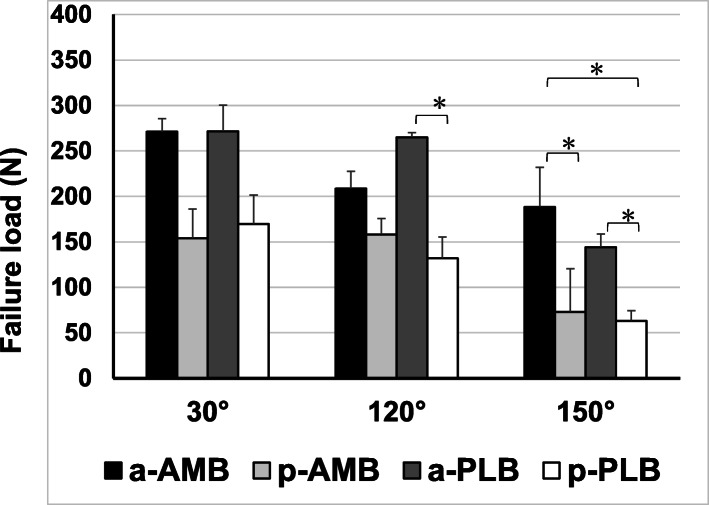
Failure loads of the four groups at the three knee flexion angles. There were no significant differences in the failure loads among the four groups in the extension position (30° of knee flexion). Conversely, there were significant differences between the a-PLB and p-PLB groups at 120°, the a-AMB and p-AMB groups at 150°, and the a-PLB and p-PLB groups at 150°. PLB, posterolateral bundle; AMB, anteromedial bundle

**Fig. 5:**
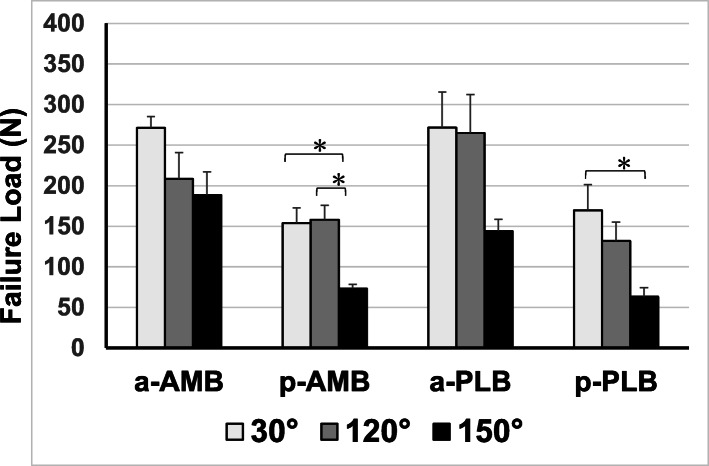
Change in failure load with knee flexion among the four insertion areas. The failure loads of all the insertion areas decreased with knee flexion. Although there was no significant difference across all the knee flexion angles in the a-AMB and a-PLB groups, which had direct insertions, there was a significant difference between the extension and flexion positions in the p-AMB and p-PLB groups. PLB, posterolateral bundle; AMB, anteromedial bundle

**Fig. 6:**
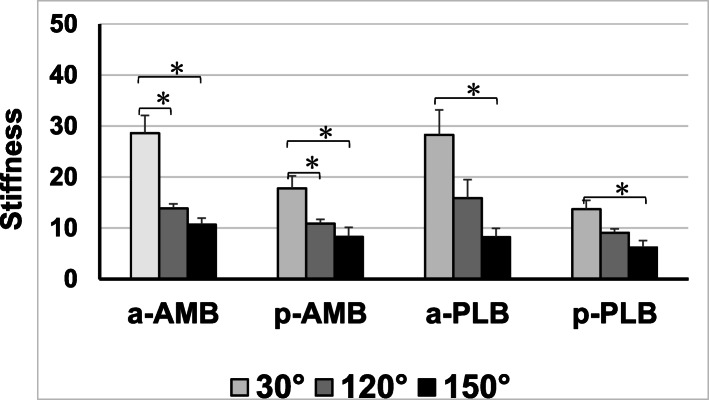
Changes in stiffness with knee flexion among the four insertion areas. Stiffness of each insertion site decreased with knee flexion

All specimens failed at the femoral side of the ACL, and there was no bone fracture or avulsion fracture during the pull-out testing. There were no significant differences in the failure loads between the four groups in terms of extension position (30° of knee flexion) (Fig. 4). Conversely, there were significant differences between the a-PLB and p-PLB groups at 120° (*p*=0.035), the a-AMB and p-AMB groups at 150° (*p*<0.001), and the a-PLB and p-PLB groups at 150° (*p*=0.014). The failure loads of all the insertion areas decreased with knee flexion (Fig. 5). Although there was no significant difference across all the knee flexion angles in both the a-AMB and a-PLB groups, which had direct insertions, there was a significant difference between the extension and flexion positions in the p-AMB and p-PLB groups. Moreover, the stiffness of each insertion site decreased with knee flexion (Fig. 6).

## Discussion

This study showed no significant differences in failure loads between the anterior portion (direct insertion) and the posterior portion of the ACL femoral insertion site (mainly consisting of indirect insertion) in 30 degrees of flexion. This result agrees with that of a previous biomechanical study on the femoral posterior fan-like extension of the ACL [13]. This implies that the indirect insertion of the ACL [7], the so-called fan-like extension fibers [8], significantly contributes to ACL strength. Our study also showed that the failure load of the ACL decreased with knee flexion, especially in the posterior portion of the ACL. Differences in the decreasing degree of the failure load may be due to ACL insertion type [17, 18]. To the best of our knowledge, this is probably the first report on this finding.

There are two types of tendon and ligament insertions into the bone; these include direct and indirect insertions or fibrocartilaginous and fibrous enthesis, according to the type of tissue present at the attachment site [17, 19]. Indirect insertion is a common form of bone insertion; tendons that attach to the diaphyses of long bones are an example [18]. Superficial fibers insert into the periosteum and deep fibers insert directly into the bone via perforating collagen fibers, known as Sharpey fibers. Direct insertion is characterized by the presence of fibrocartilage at the tendon-bone interface; this is typical of epiphyses and apophyses [18]. Direct insertions have four distinct zones: zone 1, pure dense fibrous connective tissue; zone 2, uncalcified fibrocartilage; zone 3, calcified fibrocartilage; and zone 4, bone [20, 21]. These four transitional zones allow for force dissipation and reduce stress concentration. The direction of forces applied from the ligament to the bone may contribute to the ligament insertion site morphology.

Specimen orientation and the complex anatomy of the ACL may play an important role in both, ultimate load and failure modes in tensile testing [14]. The anatomical orientation along the axis of the ACL increases ultimate load, whereas the non-anatomical orientation decreases ultimate load. In younger specimens, the ultimate load decreased from 2160 ± 157 N to 1602 ±167 N (74.2%). The knee flexion angle and displacement rate also affect the rupture pattern and the ultimate load of the ACLs. Based on a biomechanical study using rabbits [22], the majority of ACLs tested failed by avulsion at 45° flexion; the reverse was true for ACLs tested at 90°, where the majority of failures were fiber pull-outs. The ultimate load of the ACL decreased with knee flexion by approximately 30% compared to those of the 0° and 90° knee flexion angles. These results are consistent with the findings from this study.

We used porcine knees, which are anatomically different from the human knee. Although the porcine ACL tibial insertion site is clearly divided into two bundles macroscopically by the anterior attachment of the lateral meniscus, it is composed of the AMB and PLB; these are similar to those of the human knee [23, 24]. In addition, our histological study showed that the porcine ACL femoral insertion site consisted of direct and indirect insertions in the anterior and posterior portions, respectively; this histological structure is also similar to that of humans [6, 7]. Therefore, the porcine knee is considered to be the preferred model for experimental biomechanical studies [25], since it can be matched by size and age [13]. Human cadaver knees are difficult to obtain at younger ages, at which ACL injuries occur.

The tensile strengths of a 3-mm square attachment of the ACL were assessed in this study, because our pilot study showed that tensile testing of the entire ACL sometimes causes a fracture of the femoral condyle. If the medial femoral condyle is not resected, fracture may not occur; however, it is difficult to accurately identify the ACL femoral attachment site without resection of the medial condyle. Therefore, we divided the ACL insertion site into 4 areas and measured the strength of the 3-mm square attachment of each area. We believe that this method allowed us to evaluate the strength of each area according to the knee flexion angle.

This study has several limitations. As previously mentioned, porcine knees are anatomically different from human knees. As it is difficult to obtain fresh young cadaver knees, it will be impossible to prepare a sufficient number of specimens for statistical power. In addition, amputated elderly knees are not suitable for biomechanical studies, because of poor bone and soft tissue quality. Although our study changed the knee flexion angles for tensile testing, a single displacement rate of 0.33 mm/s [16] was used in this study. Changing the displacement rate in tensile testing will result in a different tensile load [22]. Although this study showed a significant role of the posterior portion in knee extension, it should be noted that this does not directly suggest the ideal position of the femoral tunnel.

## Conclusions

Although the failure load of the posterior portion decreased significantly in the knee flexion position, it (mainly consisting of indirect insertion) plays a significant role against anterior tibial load in the knee extension position; this appears to be related to the characteristics of the insertion site. Reflecting the complex structure and function of the ACL, this study showed that the failure load of the femoral insertion site varies with differences in positions and knee flexion angle.

## Data Availability

Not applicable.

## Abbreviations

ACL: Anterior cruciate ligament
DOF: Degree of freedom
AMB: Anteromedial bundle
PLB: Posterolateral bundle
FATC: Femur-ACL-tibia complexes
